# The dark side of precrastination: exploring the psychological burdens of being too early

**DOI:** 10.3389/fpsyg.2025.1698978

**Published:** 2025-12-03

**Authors:** Christopher Gehrig, Philipp Yorck Herzberg

**Affiliations:** Helmut Schmidt University/University of the Federal Armed Forces, Hamburg, Germany

**Keywords:** precrastination, chronic stress, compulsive personality characteristics, neuroticism, personality traits, precrastination scale

## Abstract

In today’s fast-paced world, where efficiency and immediacy are highly valued, one central question remains largely overlooked: When and why does early and swift action become a burden? This study focuses on the phenomenon of precrastination—the urge to complete tasks as early as possible—and sheds light on its “dark side.” Building on a recently proposed three-dimensional model of precrastination, we empirically examined the associations between anxiety- and compulsion-driven precrastination, chronic stress, and compulsive personality characteristics. In an online sample of adults (*N* = 200), anxiety-based precrastination was significantly associated with chronic stress, while compulsion-based precrastination correlated significantly with compulsive personality traits. Both subscales predicted their respective psychological correlates. These findings offer the first differentiated evidence that precrastination is not merely a sign of productivity but may also reflect maladaptive coping mechanisms. This opens a new perspective on a widely overlooked everyday behavior—and on the psychological costs that may come with acting too soon.

## Introduction

Today’s society is characterized by multitasking, fast-paced living, and swift task completion (Schaudy, 2018). Bills are paid immediately upon receipt rather than waiting, assignments are submitted early even when there’s time for review, and shopping involves carrying products through the entire store rather than taking them to the checkout on the way (Rosenbaum and Dettling, 2023; Schaudy, 2018). Such swift actions aren’t limited to everyday tasks. They also extend to more significant decisions, such as rushing into surgery without seeking a second opinion or making hasty political moves (Rosenbaum and Dettling, 2023). Not only in German culture, but also in many others, there is a proverb that reflects the value of such behavior: ‘The early bird catches the worm.’ It emphasizes the importance of early action and timely task completion as a proactive approach. In scientific terms, this behavior is referred to as “precrastination.” Precrastination can be described as the tendency to start or finish tasks early, even if it involves additional effort (Rosenbaum et al., 2014). This phenomenon has received relatively little research attention. Although precrastination was initially observed as a byproduct in a laboratory study at Pennsylvania State University (Rosenbaum et al., 2014), it was later systematically investigated in a series of behavioral experiments. In these studies, participants were asked to pick up and carry one of two buckets placed along a walking path, one closer to the starting point and one farther away, to an end point at the end of the course. Surprisingly, most participants chose the nearer bucket, thereby increasing their physical effort in order to complete the task sooner. This behavior was interpreted as a strategy to reduce cognitive load—the mental effort associated with keeping the task active in working memory (Rosenbaum et al., 2019). Subsequent research extended these findings beyond motor tasks to cognitive and motivational domains, suggesting that precrastination represents a general tendency to act early to relieve mental tension or uncertainty (Fournier et al., 2018a; Fournier et al., 2018b; Rosenbaum and Dettling, 2023).

More recently, several studies have expanded the investigation of precrastination beyond basic cognitive or motor paradigms to more complex and ecologically valid contexts. For instance, Adachi and Adachi (2024) examined precrastination in everyday decision-making, Guo et al. (2025) explored its implications for workplace functioning, and Husain et al. (2025) investigated its associations with stress regulation and well-being. Together, these studies (see also Fox et al., 2025) demonstrate a growing recognition of precrastination as a multifaceted construct that extends beyond simple efficiency-based behavior. Building on this expanding research, the present study focuses specifically on the psychological burdens of precrastination—investigating how distinct motivational forms of early action (functional, anxiety-driven, and compulsive) relate to stress and compulsive tendencies. This approach aims to complement and extend previous work by highlighting the potential burdens associated with seemingly efficient task initiation.

Initially, precrastination was regarded as the opposite of procrastination, which is well-researched today with numerous studies and measurement tools (e.g., Beutel et al., 2016; Boysan and Kiral, 2017; Engberding et al., 2017; Fournier et al., 2018a, 2018b; Kim et al., 2017; Schouwenburg, 1994, 1995; Steel and Klingsieck, 2016; Patzelt and Opitz, 2014).

Since precrastination is considered the opposite of procrastination, it has received less attention; measurement tools designed for procrastination tend to capture precrastination as well, and related research often addresses it only tangentially. However, recent studies (Gehrig et al., 2023) have shown that precrastination must be understood as a distinct construct. Although it contrasts with procrastination, it must be considered separately, with its own advantages and disadvantages (Gehrig et al., 2023). Moreover, precrastination can be distinguished from related constructs such as impulsivity and urgency (Whiteside and Lynam, 2001; Cyders and Smith, 2008). While impulsivity and negative urgency describe spontaneous, emotion-driven, or reward-oriented behavior, precrastination reflects a premature yet goal-directed tendency to act quickly to reduce cognitive tension or uncertainty. Thus, precrastination is not characterized by a lack of self-control, but rather by an excessive striving for control and completion. Furthermore, we explicitly distinguish precrastination from the construct of negative urgency. Although both constructs involve a tendency toward rapid action under internal tension, their underlying processes differ. Negative urgency refers to rash, emotion-driven behavior performed to alleviate acute emotional distress or negative affect (Cyders and Smith, 2008). In contrast, precrastination represents a structured and premature form of action aimed at reducing cognitive load and the emotional tension that accompanies uncertainty or anxiety through early task completion. Thus, while both may serve an emotion-regulatory function, negative urgency reflects impulsive and reactive behavior, whereas precrastination reflects controlled and anticipatory over-regulation.

More recently, research has begun to explore the personality correlates and the potentially adaptive or maladaptive consequences of precrastination. Previous studies by Gehrig et al. (2023) and Gehrig and Herzberg (2025) indicated that precrastination is associated with conscientiousness and a heightened need for order and control—traits that may promote efficient behavior but also increase vulnerability to stress and overcontrol. Building on this line of work, the present study aims to clarify the psychological costs of precrastination by examining its associations with chronic stress and compulsive personality characteristics.

Contrary to popular belief, the opposite of precrastination is not procrastination; it is rather a “normal working style.” If precrastination is seen as a distinct construct, it exhibits both positive and negative aspects, much like procrastination. Initial indications that precrastinatory behavior can be viewed as both beneficial and detrimental to individuals are found in studies by Gehrig et al. (2023) and Gehrig and Herzberg (2025), which highlight associations with personality facets resulting in both positive and detrimental outcomes. By adjusting the above quote, it becomes clear what this work aims to achieve: “The early bird catches the worm but falls exhausted from the branch in the evening.” This phrase suggests that precrastinatory behavior, as indicated in the literature, may not only have positive outcomes but can also be considered detrimental—the Dark Side of Precrastination.

The measurement instrument developed by Gehrig and Herzberg (2025) includes three dimensions. The first is functional precrastination, which can be considered the “positive” form of precrastination. The other two dimensions capture precrastination driven by compulsion and fear—forms of “harmful precrastination.” This tendency to precrastinate due to anxiety or a sense of pressure is the central focus of this work. Functional precrastination describes the original construct identified by Rosenbaum et al. (2014) in their laboratory study at Penn State University. It refers to the tendency to start or complete tasks as early as possible, even when this involves unnecessary or increased effort. As mentioned earlier, precrastination was initially understood as the functional opposite of the well-researched concept of procrastination (Steel, 2007; Schouwenburg, 1995). While procrastination is associated with delaying behavior, dysfunctional emotion regulation, and reduced performance (Sirois and Pychyl, 2013), precrastination was considered a sign of efficiency and goal orientation. However, recent research suggests that precrastination is not inherently adaptive (Gehrig et al., 2023). Rather like procrastination, it appears to be driven by both functional and dysfunctional motivations.

Precrastination driven by fear can be understood as a form of anticipatory action aimed at avoiding negative emotional states—such as stress or fear of failure. This may manifest in the immediate initiation of tasks out of concern that they may not be completed in time before the deadline (Gehrig and Herzberg, 2025). The transactional model of stress by Lazarus and Folkman (1984) provides one interpretative framework for understanding this process: an upcoming task is appraised in the primary evaluation as potentially threatening—such as in terms of possible failure or time pressure. The chosen coping strategy is to start the task immediately in order to quickly reduce the perceived or anticipated threat (Lazarus and Folkman, 1984).

However, this model should not be seen as the sole explanation for precrastinatory behavior; alternative mechanisms, such as perfectionistic overcontrol and anxiety-driven avoidance, may likewise contribute. While this behavior may help reduce emotional distress in the short term, it can become dysfunctional in the long run—especially when the need for control or immediate task completion becomes chronically excessive, thereby maintaining or even increasing stress levels (Lazarus and Folkman, 1984). This behavior becomes particularly problematic when precrastinative action turns into an obligation, regardless of its actual necessity or usefulness. In such cases, the behavior closely resembles cognitive avoidance strategies, as described in the context of generalized anxiety disorders (Dugas and Robichaud, 2007). In these cases, quick action is often intended to (supposedly) control future events, but in the long term, it remains ineffective and emotionally burdensome (Borkovec et al., 2004). Previous research has suggested that precrastination may serve as a maladaptive coping strategy aimed at reducing anxiety-related tension through premature task completion. This notion parallels avoidance-based coping mechanisms, which provide short-term relief at the expense of long-term stress regulation. Neuroticism—a personality trait closely associated with anxiety and stress vulnerability—is a well-established transdiagnostic risk factor for various forms of psychopathology (Lahey, 2009). Moreover, procrastination-related behaviors have been linked to maladaptive emotion regulation strategies that prioritize short-term relief over adaptive problem-solving (Sirois and Pychyl, 2013). These findings support the conceptualization of precrastination as an overcontrolled, anxiety-related coping tendency rather than a purely efficiency-driven behavior.

In addition to the fear component, precrastination can also be driven by a rigid internal sense of obligation. In such cases, it is referred to as precrastination due to compulsion—a behavior not triggered by an acute threat, but rather by a compulsively felt need to complete tasks immediately (see Gehrig and Herzberg, 2025). This behavioral tendency shares conceptual similarities with characteristics observed in obsessive-compulsive personality styles, such as an exaggerated sense of responsibility, rigid control needs, and perfectionism (Salkovskis, 1985; Frost and Steketee, 2002; World Health Organization, 1993). Within cognitive-behavioral models, such internally driven obligations are viewed as dysfunctional cognitions that can promote repetitive or rigid behaviors aimed at reducing discomfort, even in the absence of external pressure (Obsessive Compulsive Cognitions Working Group, 2005). Although the concept of working-memory offloading has been discussed in connection with precrastination, it should be understood as a resulting cognitive function of early task initiation rather than as its primary motivational cause. Research suggests that individuals with compulsive tendencies may experience increased cognitive load and reduced working memory flexibility (Purcell et al., 1998a, 1998b) and may therefore engage in premature task execution as a secondary strategy to relieve this cognitive strain (Rosenbaum et al., 2019; Fournier et al., 2018a; Fournier et al., 2018b). Thus, while fear-based and compulsion-based precrastination both lead to early task initiation, they differ in their primary motivational dynamics: fear-based precrastination is driven by anxiety reduction and emotional avoidance, whereas compulsion-based precrastination stems from rigid control needs and perfectionistic overcontrol. This conceptual distinction clarifies the multidimensional nature of precrastination and provides a theoretical foundation for differentiating functional from maladaptive forms. This conceptual overlap suggests that individuals high in compulsive traits or neuroticism (Samuels et al., 2000; Gehrig et al., 2023; Gehrig and Herzberg, 2025) may engage in early task completion as a self-regulatory mechanism to reduce internal pressure, rather than as an efficient problem-solving strategy. Taken together, these findings indicate a subclinical pattern of overcontrol rather than clinical OCD symptomatology.

It is important to note that the present study does not assess clinical symptoms of obsessive-compulsive disorder but rather personality tendencies associated with orderliness and control. The “careful–compulsive” subscale of the PSSI (Kuhl and Kazén, 2009) captures non-pathological personality styles that may resemble obsessive-compulsive features at high levels but are dimensional in nature. Accordingly, all variables in this study were treated as continuous measures to reflect dispositional tendencies rather than categorical disorders. This approach aligns the measurement strategy with the study’s aim to investigate psychological burdens linked to subclinical traits of precrastination.

Overall, it becomes clear that precrastination is a construct that requires a differentiated perspective. While functional precrastination may, in certain contexts, be associated with efficiency and organization (Rosenbaum et al., 2014), fear- and compulsion-based forms carry significant risks for psychological well-being (Gehrig and Herzberg, 2025). They are not merely variations of rapid task completion but may instead reflect deeper emotional or cognitive dysregulation (Purcell et al., 1998a, 1998b; Frost and Steketee, 2002). Distinguishing between these dimensions is not only theoretically important but also crucial for the prevention and intervention of psychological strain (Obsessive Compulsive Cognitions Working Group, 2005; Samuels et al., 2000). Thus, the aim of this study is twofold: first, to investigate the relationship between the precrastination scales and chronic stress as well as compulsive personality characteristics; and second, to further validate the two burden-related scales developed by Gehrig and Herzberg (2025). Accordingly, the following research question was formulated: How are the precrastination scales related to chronic stress and compulsive personality characteristics, and to what extent can empirical support for the validity of the two disorder-specific scales proposed by Gehrig and Herzberg (2025) be demonstrated?

Based on this, the following hypotheses were proposed: (1) There is a positive relationship between fear-based precrastination and chronic stress. (2) There is a positive relationship between compulsive precrastination, and compulsive personality characteristics. (3) The disorder-specific subscales show differential associations with chronic stress and compulsive personality characteristics, consistent with their conceptual distinctions.

## Method

### Sample

The data were collected at the end of 2024 using the online survey platform SoSci Survey (Leiner, 2019a). The initial dataset comprised 273 participants. To ensure data quality and validity, we followed the data-cleaning standards recommended by Leiner (2019b) using SoSci Survey’s internal quality control indices. Participants were excluded if they showed implausibly short completion times (less than one-third of the median completion time) or received high penalty scores indicating non-attentive responding. Based on these criteria, 73 cases were removed, resulting in a final sample of 200 participants. The remaining dataset was then inspected for completeness and plausibility. No extreme values or implausible response patterns were identified, and all valid cases were retained for analysis. This procedure ensured high data quality and reproducibility. The majority of the sample consisted of young German adults. Out of the 200 participants, 94 identified as women (47%) and 106 as men (53%), with a mean age of 26.6 years (SD = 9.48). Participation was voluntary, and electronic informed consent was obtained from all participants. No personally identifying information was collected, ensuring pseudonymization and data protection in line with ethical research standards. Eligibility required a minimum age of 18 years and sufficient German language proficiency. Additional details regarding the sample description, calculations, tables, as well as access to the dataset used in this study, are available via the Open Science Framework repository (Foster and Deardorff, 2017) (https://doi.org/10.17605/OSF.IO/J3S4D).

### Instruments

Precrastination was measured using the *Facets of (Mal)adaptive Precrastination Scale (FIPS)* (Gehrig and Herzberg, 2025), a recently developed instrument that assesses the construct across three distinct dimensions: functional precrastination, fear-based precrastination, and compulsive precrastination. The *functional precrastination* subscale (13 items) measures the general tendency to initiate tasks immediately, regardless of contextual demands. Internal consistency was excellent (Cronbach’s *α* = 0.96). An example item illustrating this scale is: ‘*I prefer not to delay tasks’.* The *fear-based precrastination* subscale (10 items) captures premature task initiation aimed at avoiding aversive emotional states, such as overwhelm or loss of control, and also demonstrated high internal consistency (*α* = 0.95). An example of this would be: ‘*I fear that too many tasks will overwhelm me’*. The *compulsive precrastination* subscale (14 items) reflects a compulsive inner urge to begin tasks immediately, independent of external pressure, and showed strong reliability (α = 0.92). An item exemplifying this scale is: *‘I cannot stand not starting a task immediately’*. All items were rated on a 5-point Likert scale ranging from 1 (*does not apply at all*) to 5 (*fully applies*), with higher scores indicating greater levels of the respective precrastination type. Construct validity has been confirmed through factor analyses and significant correlations with personality and opposing measures.

Chronic stress was assessed using the Screening Scale for Chronic Stress (SSCS; Schulz et al., 2004), a short form of the Trier Inventory for Chronic Stress (TICS). The SSCS includes 12 items derived from the original 57-item version and measures general chronic stress across five domains: chronic worry, work-related overload, social overload, excessive demands, and lack of social recognition. For conceptual clarity, the acronym *SSCS* is used consistently throughout this manuscript when referring to this measure. Responses were provided on a 5-point scale (0 = *never* to 4 = *very often*), and item scores were summed to obtain total scores, with higher scores reflecting greater chronic stress. The SSCS demonstrated excellent internal consistency (Cronbach’s *α* = 0.91). Construct validity has been confirmed through factor analyses and significant correlations with other stress-related and personality measures.

Compulsive personality style was measured with the *“careful–compulsive”* (ZW) subscale of the *Personality Style and Disorder Inventory (PSSI)* (Kuhl and Kazén, 2009). The PSSI is a self-report instrument published by Hogrefe and designed to assess non-pathological personality styles rather than clinical disorders. It correspond to personality disorders described in the *DSM-IV-TR* (American Psychiatric Association, 2000). The ZW subscale comprises 10 items rated on a 4-point scale (0 = *does not apply at all* to 3 = *fully applies*), capturing characteristics such as precision, orderliness, and structured task completion. While high scores may resemble obsessive-compulsive features, the scale captures dimensional personality tendencies associated with orderliness and control rather than symptoms of obsessive-compulsive disorder. The scale demonstrated good internal consistency (Cronbach’s *α* = 0.85). Item-total correlations ranged from 0.43 to 0.74. The scale’s construct validity has been supported by factor analyses and significant correlations with psychosomatic symptoms and Big Five traits.

Personality was measured using the *Big Five Inventory-10 (BFI-10)* (Rammstedt and John, 2007), a brief instrument based on the Big Five model. The scale includes 10 items, with two items per trait: extraversion, agreeableness, conscientiousness, neuroticism, and openness to experience. Items were rated on a 5-point Likert scale (1 = *does not apply at all* to 5 = *fully applies*). The BFI-10 shows acceptable test–retest reliability (rtt = 0.58–0.84) and strong content, factorial, and construct validity. The latter is evidenced by high correlations with the NEO Personality Inventory (NEO-PI-R; Costa and McCrae, 1992) and low correlations with non-corresponding dimensions.

All instruments used in this study are well-established and psychometrically validated measures. The Screening Scale for Chronic Stress (SSCS; Schulz et al., 2004) and the Personality Styles and Disorders Inventory (PSSI; Kuhl and Kazén, 2009) are standardized instruments published by Hogrefe. The reliability coefficients observed in the present sample (Cronbach’s *α* = 0.85–0.96) indicate good to excellent internal consistency across all scales. Moreover, the constructs have demonstrated solid factorial and convergent validity in previous validation studies, supporting their suitability for investigating personality- and stress-related processes in non-clinical populations.

### Data analysis

All results presented in this paper were calculated using the statistical software programs SPSS (IBM Corp, 2017) and R (R Core Team, 2018). Prior to analysis, the data were imported from SoSci Survey, negatively worded items were recoded, and total scores and mean values for the scales were computed. Descriptive statistics were used to analyze sociodemographic characteristics such as age, gender, and educational background.

The statistical assumptions for the analyses were checked, including the identification of outliers, testing for the normal distribution of residuals, homoscedasticity, linearity, and multicollinearity. All assumptions were met. Scale reliability was assessed using Cronbach’s α. Additionally, a correlation matrix was computed to examine bivariate relationships.

To test the hypotheses, multiple regression analyses were conducted, with personality included as a control variable. Gender was dummy coded (0 = male, 1 = female) and included as an additional control variable in all regression analyses.

In line with the study’s theoretical framework, stress (SSCS) and compulsive tendencies (PSSI) were treated as outcome variables, as the primary aim was to examine how different forms of precrastination contribute to psychological strain rather than result from it.

Furthermore, the data were explored for potential interactions between key variables. Specifically, the interaction between anxiety-related precrastination and neuroticism was tested to explore whether emotional instability amplifies stress-related responses. For theoretical comparison, a parallel exploratory model tested the interaction between compulsive precrastination and conscientiousness.

## Results

### Descriptive statistics and gender differences

Descriptive statistics for all study variables, including means and standard deviations are reported in the OSF repository (Supplementary Table S4; “Descriptive Statistics”). Independent samples *t*-tests were conducted to examine potential gender differences. Results indicated that women reported significantly higher levels of anxiety-related precrastination [*t*(198) = −4.55, *p* < 0.001], compulsive precrastination [*t*(198) = −2.48, *p* = 0.015], chronic stress [*t*(198) = −3.51, *p* = 0.001], neuroticism [*t*(198) = −5.30, *p* < 0.001], and conscientiousness [*t*(198) = −3.00, *p* = 0.003] compared to men. No significant gender differences were found for functional precrastination, compulsive personality traits, or the remaining Big Five dimensions (*p*s > 0.05). These findings are consistent with prior research indicating higher stress and anxiety-related tendencies among women.

### Reliability

The reliability of all scales was examined and compared to the reliability coefficients reported in the respective manuals. This served to assess how consistently the results of the present study can be interpreted. Table 1 presents all reliability coefficients. All values fall within the good to excellent range, with only minimal deviations from the values reported in the manuals. Thus, it can be assumed that the scales used in this study demonstrate satisfactory reliability within the current sample.

**Table 1:** Cronbach’s alpha.

	Cronbach’s alpha		Number of items
	Current sample	original tool	
Functional precrastination	0.93	0.96	13
Precrastination out of fear	0.87	0.95	10
Precrastination out of compulsion	0.95	0.92	14
SSCS	0.92	0.91	12
PSSI	0.77	0.85	10

SSCS = Screening Scale for Chronic Stress (Schulz et al., 2004); PSSI = Personality Styles and Disorders Inventory (Kuhl and Kazén, 2009); *n* = 200.

### Correlations

In the next step of the analysis, correlations between the central variables were calculated to systematically examine their bivariate relationships. A complete overview of all correlations is presented in Table 2; additional information, including means and standard deviations, is available on OSF. Furthermore, a full correlation matrix including all study variables (SSCS, PSSI, FIPS subscales, and Big Five) as well as a visual correlogram (heatmap) are provided in the OSF repository to enable a transparent evaluation of all intercorrelations. Overall, the results confirm the associations reported in previous research, both in terms of direction and theoretical meaning. These findings provide additional empirical support for the assumed theoretical relationships and contribute to the validation of the scales employed in this study. Particular attention was given to the scales *Precrastination due to Anxiety* and *Precrastination due to Compulsion*, which are central to the research question.

**Table 2:** Correlations between the facets of the questionnaire, the PSSI, the SSCS and the personality dimensions.

	Functional precrastination	Precrastination out of fear	Precrastination out of compulsion
Functional precrastination			
Precrastination out of fear	−0.14		
Precrastination out of compulsion	0.60*	0.21*	
SSCS	−0.09	0.65***	0.28*
PSSI	0.40*	0.07	0.38**
Extraversion	0.06	−0.30**	0.04
Agreeableness	0.09	−0.04	0.17*
Conscientiousness	0.57***	−0.06	0.44*
Neuroticism	−0.07	0.69***	0.18**
Openness	−0.11	0.13	−0.02

SSCS = Screening Scale for Chronic Stress (Schulz et al., 2004); PSSI = Personality Styles and Disorders Inventory (Kuhl and Kazén, 2009); *n* = 200, Signif. codes: 0 ‘***’, 0.001 ‘**’, 0.01 ‘*’, 0.05 ‘.’, 0.1, 1. These correspond to the default significance codes used in R.

The *Precrastination due to Anxiety* scale showed significant positive correlations with the stress scale from the Screening Scale for Chronic Stress (SSCS) (*r* = 0.65; *p* < 0.01) and the personality dimension Neuroticism (*r* = 0.69; p < 0.01). These results suggest that precrastinative behavior driven by anxiety is closely linked to heightened stress levels and increased emotional reactivity.

The second specific facet, *Precrastination due to Compulsion*, also demonstrated the expected associations. It correlated significantly with the compulsivity scale of the Personality Style and Disorder Inventory (PSSI) (*r* = 0.38; *p* < 0.01), indicating conceptual proximity between the constructs. Additionally, a weaker yet still significant correlation with Neuroticism was observed (*r* = 0.18; *p* < 0.01), which aligns with theoretical assumptions, as compulsive tendencies are often accompanied by heightened emotional sensitivity.

As reported in earlier studies, the *functional Precrastination* scale showed a significant positive correlation with Conscientiousness (*r* = 0.57; *p* < 0.01). This finding suggests that individuals with high levels of conscientiousness—those who tend to behave in an organized, structured, and goal-oriented manner—are more likely to begin or complete tasks at an early stage. This supports the notion that precrastination is not exclusively a dysfunctional behavior, but can also reflect functional aspects of efficient action regulation. Overall, these correlation results support the theoretical differentiation of the precrastination construct.

### Regression models

To further explore the relationships between different facets of precrastination and psychological distress, and to test the proposed hypotheses, two multiple regression analyses were conducted. An overview of the model results can be found in Table 3. The goal was to examine the predictive relevance of the three subscales from the newly developed Precrastination Assessment by Gehrig and Herzberg (2025)— *Functional Precrastination*, *Precrastination due to Anxiety*, and *Precrastination due to Compulsion*—in interaction with core personality traits (Big Five, included as control variables) regarding chronic stress and compulsive behavior. In addition, we tested the incremental variance explained by models including the FIPS subscales compared to models without them (see Table 3).

**Table 3:** Multiple regression models on PSSI and SSCS.

	SSCS (stress)			PSSI (compulsion)		
	*B*	*(SE)*	*p*	*B*	*(SE)*	*p*
Functional precrastination	−1.64	0.93	0.049*	0.50	0.51	0.331
Precrastination out of fear	4.70	0.97	<0.001***	0.89	0.54	0.089.
Precrastination out of compulsion	3.80	0.93	<0.001***	0.90	0.52	0.045*
Conscientiousness	−1.29	0.74	0.081.	2.39	0.41	<0.001***
Neuroticism	2.80	0.70	<0.001***	−0.52	0.39	0.187
Extraversion	−0.15	0.53	0.770	−0.32	0.29	0.275
Agreeableness	−0.81	0.68	0.243	−0.48	0.38	0.206
Openness	−0.40	0.50	0.437	−0.52	0.28	0.061.
Explained variance (*R*^2^)	0.49			0.31		

SSCS = Screening Scale for Chronic Stress (Schulz et al., 2004); PSSI = Personality Styles and Disorders Inventory (Kuhl and Kazén, 2009); *n* = 200. Signif. codes: 0 ‘***’, 0.001 ‘**’, 0.01 ‘*’, 0.05 ‘.’, 0.1, 1. Compared to restricted models without the FIPS subscales, explained variance increased significantly from *R*^2^ = 0.36 to *R*^2^ = 0.49 for stress models (Δ*R*^2^ = 0.13, *p* < 0.001), and from *R*^2^ = 0.27 to *R*^2^ = 0.31 for compulsion models (Δ*R*^2^ = 0.04, *p* < 0.05).

In the first model, chronic stress served as the dependent variable. The analysis revealed that *Precrastination due to Anxiety* was a significant predictor of chronic stress. This suggests that individuals who engage in early task completion to avoid internal tension and uncertainty report considerably higher levels of stress. Similarly, *Precrastination due to Compulsion* showed a significant positive effect, indicating that this pattern is also associated with elevated psychological strain—likely due to the cognitive demands of maintaining control and order. Interestingly, functional precrastination was negatively associated with stress, which may indicate a tendency toward lower perceived stress among individuals engaging in early task initiation for organizational reasons rather than anxiety or compulsion. Given the small effect size, this finding should be interpreted with caution and does not imply a causal stress-reducing effect. Additionally, as theoretically expected, Neuroticism emerged as a significant positive predictor of chronic stress, underlining the emotional instability and stress vulnerability of highly neurotic individuals. Conscientiousness showed a marginal negative association with stress, which could point to a protective effect of structured, organized personality traits. Moreover, Gender (0 = male, 1 = female) was included as a control variable but did not significantly predict chronic stress, *β* = 0.40, *t*(190) = 0.35, *p* = 0.73. The inclusion of gender did not alter the significance or direction of any reported effects. Overall, the model explained 49% of the variance in chronic stress (*R*^2^ = 0.49), indicating strong model fit. An ANOVA comparing the model including the FIPS subscales with a baseline model without them showed a significant increase in explained variance (Δ*R*^2^ = 0.13, from 0.36 to 0.49, *p* < 0.001). Based on these results, the first hypothesis is supported: there is a positive relationship between *Precrastination due to Anxiety* and chronic stress (see Table 3).

In the second regression model, compulsive behavior was entered as the criterion. Here, *Precrastination due to Compulsion* emerged as a significant positive predictor of compulsive tendencies. This finding suggests that individuals who complete tasks prematurely due to rigid internal demands for control exhibit higher levels of compulsive behavior. *Precrastination due to Anxiety* also showed a positive association with compulsivity, though this only reached trend-level significance. No significant relationship was found for *functional Precrastination*. Notably, Conscientiousness was positively associated with compulsive behavior, suggesting that while structured and orderly behavior is typically adaptive, it may, when overexpressed, be linked to rigid, compulsive tendencies. Additionally, Gender, included as a control variable, was not a significant predictor of compulsive behavior, *β* = −0.26, *t*(190) = −0.40, *p* = 0.69. The inclusion of gender did not change the overall pattern of results. The overall model accounted for 31% of the variance in compulsive behavior (*R*^2^ = 0.31), indicating a solid explanatory model. Here, too, an ANOVA revealed a significant incremental increase compared to the baseline model without the FIPS subscales (Δ*R*^2^ = 0.04, from 0.27 to 0.31, *p* < 0.05). Accordingly, the second hypothesis is also supported: *Precrastination due to Compulsion* is positively related to compulsive behavior (see Table 3). Across both regression models, the inclusion of gender did not alter the pattern or strength of the observed effects, indicating that the results were robust across gender.

In summary, the present findings underscore the theoretical and practical relevance of the two disorder-specific subscales of the newly developed precrastination measure. *Precrastination due to Anxiety* emerged as a key predictor of chronic stress, while *Precrastination due to Compulsion* was closely linked to compulsive tendencies. These results support the third hypothesis, which proposed differential associations between the disorder-specific subscales and the outcome measures. The observed correlation patterns are consistent with the theoretical differentiation of the subscales, providing preliminary evidence for their conceptual consistency and theoretical distinction. Collectively, the findings align with the theoretical framework of the disorder-specific dimensions within the Precrastination Assessment (FIPS) developed by Gehrig and Herzberg (2025).

### Discriminant validity and suppression effects

To further examine the distinctiveness of the three precrastination dimensions and to clarify potential statistical artifacts, additional analyses were conducted. A comparison of the dependent correlations between Functional and Compulsive Precrastination with compulsive personality tendencies (PSSI) revealed no significant difference between the two associations (*r* = 0.40 vs. *r* = 0.38; Steiger’s *z* = 0.46, *p* = 0.64). This indicates that, at the bivariate level, both subscales share a comparable magnitude of association with compulsive personality traits.

However, the multivariate regression models demonstrated clearly distinct predictive patterns. When all three FIPS subscales were entered simultaneously, Functional Precrastination displayed a small negative coefficient for stress (*B* = −1.64, *p* = 0.078), whereas both Anxiety- and Compulsion-driven Precrastination remained robust positive predictors (*B*s = 4.70 and 3.80, both *p*s < 0.001). The negative association of the functional dimension, despite its near-zero zero-order correlation, is consistent with a statistical suppression effect rather than a true stress-reducing mechanism. Separate regression models including only one FIPS subscale at a time confirmed this interpretation: the anxiety- and compulsion-based dimensions each explained substantial variance in stress (adjusted *R*^2^ = 0.45 and 0.41, respectively), while the functional dimension did not (adjusted *R*^2^ = 0.35). Variance Inflation Factors (1.07–2.19) were well below critical thresholds, ruling out problematic multicollinearity. Together, these results confirm that the observed suppression effect is statistical in nature and does not undermine the conceptual distinction between functional, anxiety-related, and compulsive forms of precrastination. Instead, they support the discriminant validity and theoretical soundness of the three-dimensional structure proposed in the FIPS model.

### Additional analysis

In addition to hypothesis testing, exploratory analyses were conducted to examine potential interaction effects between key variables. This was guided by theoretical considerations suggesting that anxiety-related precrastination may interact with neurotic traits to intensify perceived stress. A particular focus was placed on investigating whether the interaction between Precrastination due to Anxiety and Neuroticism may increase perceived stress levels. Prior to this, model comparisons using ANOVA were carried out to determine the usefulness of including specific control variables. The simpler model—limited to the essential predictors—showed a better fit and was therefore retained.

**Figure 1:**
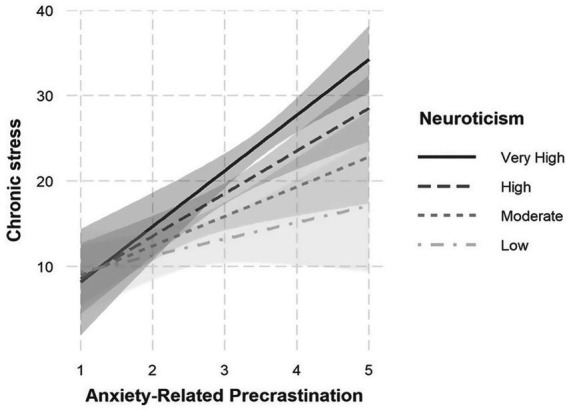
Interaction between anxiety-related precrastination and neuroticism on chronic stress.

Within this model, a significant interaction effect between Precrastination due to Anxiety and Neuroticism emerged (*B* = 1.69, SE = 0.61, *p* = 0.007). This interaction suggests that the relationship between anxiety-driven precrastination and stress is moderated by the level of neuroticism. The interaction is illustrated in Figure 1. As shown in the figure, the positive association between anxiety-related precrastination and chronic stress was strongest at high levels of neuroticism, weaker at moderate levels, and absent at low levels of neuroticism. In other words, individuals who not only tend to complete tasks prematurely out of fear of not finishing on time but also exhibit a high degree of emotional instability and susceptibility to negative affect, report particularly high levels of stress. This pattern may suggest that for emotionally unstable individuals, the short-term relief strategy of acting immediately could coincide with heightened stress perception, rather than effectively reducing stress in the long run. Thus, precrastinative behavior, typically intended to relieve internal tension, may in this subgroup intensify rather than alleviate psychological strain.

For comparison, an exploratory model including the interaction between compulsive precrastination and conscientiousness was also tested. This analysis yielded a trend-level effect (*B* = 0.90, SE = 0.46, *p* = 0.052), suggesting that the moderating influence of conscientiousness on compulsive tendencies may be weak or sample-specific. Hence, the significant moderation observed for neuroticism appears to be specific to anxiety-driven precrastination.

## Discussion

The aim of the present study was to investigate different facets of precrastination—particularly those driven by anxiety and compulsiveness—and their associations with chronic stress and compulsive behavior. In addition, the theoretically grounded differentiation of the construct was empirically examined. The results of the regression and correlation analyses support the relevance of this distinction: While anxiety-driven precrastination emerged as a strong positive predictor of chronic stress, compulsively driven precrastination also showed a significant yet weaker association. In contrast, functional precrastination was negatively related to stress, which may indicate a tendency toward lower perceived stress rather than a direct stress-reducing effect (cf. Rosenbaum et al., 2014; Gehrig et al., 2023). This association could reflect an underlying structured self-management style or higher levels of conscientiousness, which help individuals stay organized and maintain control in everyday tasks. In this sense, functional precrastination may co-occur with lower stress perceptions as a byproduct of adaptive personality traits, rather than as a causal mechanism.

Differential effects also emerged regarding compulsive personality traits: Most notably, compulsively driven precrastination was significantly associated with the “conscientious-compulsive” personality style (PSSI), highlighting its closeness to the compulsivity spectrum (Frost and Steketee, 2002). The positive link between conscientiousness and compulsive behavior suggests that functional personality traits may become maladaptive at their extremes (Samuels et al., 2000).

Although the correlations between functional and compulsive precrastination with compulsive personality traits were similar in size, supplementary analyses confirmed that this pattern reflects a statistical overlap rather than conceptual redundancy. The suppression effect observed in the regression model indicates that functional precrastination operates in the opposite direction once shared variance with maladaptive forms is controlled for. This supports the theoretical assumption that functional precrastination represents a distinct, adaptive form of early task initiation, whereas anxiety- and compulsion-driven tendencies reflect maladaptive control motives.

While functional and compulsive forms of precrastination share a substantial amount of variance (*r* = 0.60), this positive correlation does not imply conceptual redundancy or contradict their theoretical distinction. Both forms reflect early task initiation as a common behavioral expression but differ fundamentally in their underlying motivational dynamics. Functional precrastination is typically guided by goal-directed organization and planning tendencies, whereas compulsive precrastination stems from an excessive need for control and the avoidance of inner tension. Thus, the positive correlation likely reflects overlapping behavioral patterns rather than equivalent psychological mechanisms. Likewise, the shared association with conscientiousness does not suggest that this trait is “half adaptive and half maladaptive,” but rather that conscientiousness can function as both a resource (supporting structure and diligence) and, under conditions of rigidity or perfectionism, as a risk factor for maladaptive overcontrol.

These findings underscore that functional and compulsive precrastination represent distinct yet partially overlapping facets of the same behavioral domain, consistent with their theoretically differentiated motivational basis. This interpretation aligns with meta-analytic evidence indicating that conscientiousness can act as a “double-edged sword,” reflecting both adaptive striving and maladaptive perfectionistic tendencies when expressed excessively (Smith et al., 2019).

A particularly insightful finding came from an exploratory analysis of the interaction between anxiety-driven precrastination and neuroticism. Here, the stress-enhancing effect of anxious precrastination was significantly amplified by high levels of neuroticism. This points to a moderating role of emotional instability: For individuals with high emotional reactivity, completing tasks early does not appear to reduce stress but instead reinforces it—possibly because this strategy provides short-term relief while maintaining dysfunctional patterns in the long run.

Overall, the findings provide strong empirical support for the theoretical assumption that precrastination is a multidimensional construct that operates differently across psychological contexts. Differentiating between functional and dysfunctional motivational patterns (e.g., anxiety- or compulsion-driven vs. deliberate and early) appears not only theoretically meaningful but also practically relevant—for example, in terms of assessment and intervention strategies.

### Contribution and outlook

This study makes a contribution to the relatively understudied topic of precrastination by empirically distinguishing functional from dysfunctional forms. The findings suggest that it is not the behavior itself—completing tasks early—that is inherently problematic, but rather its motivational basis. When driven by internal pressure, anxiety, or rigid control, such behavior may contribute to psychological strain. The three-dimensional instrument developed by Gehrig and Herzberg (2025) enables a nuanced assessment of these patterns and may prove valuable for future clinical diagnostics, particularly in the context of anxiety disorders or obsessive-compulsive personality styles (Obsessive Compulsive Cognitions Working Group, 2005). The results may also inform intervention strategies, especially within cognitive-behavioral frameworks. Preventive programs aimed at stress management could benefit from addressing precrastinative tendencies in a differentiated manner. Future research should focus on longitudinal and intervention-based designs to explore the temporal dynamics of precrastinatory behavior and its modifiability. Moreover, moderating variables such as emotion regulation (Aldao et al., 2010), perfectionism, or intolerance of uncertainty offer promising avenues for further investigation.

### Limitations

Despite the informative findings, this study has several limitations. First, it employed a cross-sectional design, which does not allow for causal conclusions. It remains unclear whether precrastination contributes to psychological strain or rather reflects a response to existing stressors. Second, all data were collected through self-report measures, which may be prone to biases such as social desirability or limited self-awareness—particularly with regard to internalizing symptoms like compulsiveness or anxiety (cf. Dugas and Robichaud, 2007). Future research should incorporate objective data sources or third-party assessments to complement self-reports. Third, the sample predominantly consisted of young participants from German-speaking countries, which limits the generalizability of the findings. Cultural differences in dealing with time pressure and performance demands could influence both the expression and interpretation of precrastinatory behavior. Fourth, the construct of precrastination was assessed using a newly developed instrument which—despite its strong reliability and theoretical grounding—is still in the early stages of scientific validation. The scales have yet to be tested in clinical populations or intervention settings. Finally, it should be noted that the Anxiety-related Precrastination subscale contains several emotionally laden items (e.g., referring to fear or worry), which may have contributed to inflated correlations with affectively similar constructs such as chronic stress (SSCS) and neuroticism. This overlap in item content might partly reflect shared emotional wording rather than true conceptual overlap. Future research should refine the item wording to minimize this potential bias and verify whether the observed effects persist when emotional valence is reduced. Furthermore, while construct validity was supported through correlations with relevant personality dimensions and stress indicators, a more comprehensive convergent and discriminant validation—especially in comparison with established questionnaires on emotion regulation or action control—would be a valuable next step.

## Conclusion

The findings of this study suggest that precrastination is not a uniformly positive behavior. Rather, the psychological correlates differ substantially depending on whether tasks are completed early due to planning or due to anxiety and compulsion. Functional precrastination may be accompanied by a sense of relief, whereas dysfunctional forms are associated with increased stress and compulsive tendencies. This distinction is not only theoretically significant but also has direct implications for psychological diagnostics and interventions. It enables a more nuanced view of superficially similar behavior—such as completing tasks early—by differentiating between effective self-organization and maladaptive control.

The results further indicate that the impulse to “get things done immediately” may, in some cases, reflect not efficiency but rather inner tension or cognitive overload. At the same time, the findings emphasize the need to give more attention to the complexity of precrastinatory behavior in both research and practice. The measurement tool developed by Gehrig and Herzberg (2025) provides a solid foundation for this, allowing for a more precise assessment of distinct forms and their psychological correlates.

Overall, the study suggests that the pursuit of efficiency and control—common though it may be—may co-occur with psychological strain when driven by rigid or anxiety-based internal processes. Precrastination, therefore, deserves not only increased scholarly attention but also a central role in discussions about healthy self-regulation and sustainable work habits.

## Data Availability

The datasets presented in this study can be found in online repositories. This data can be found here: https://doi.org/10.17605/OSF.IO/J3S4D.
